# Prediction of Motion Intentions as a Novel Method of Upper Limb Rehabilitation Support

**DOI:** 10.3390/s21020410

**Published:** 2021-01-08

**Authors:** Bogusz Lewandowski, Sławomir Wudarczyk, Przemysław Sperzyński, Jacek Bałchanowski

**Affiliations:** Department of Mechanical Engineering, Wroclaw University of Science and Technology, 30-370 Wroclaw, Poland; slawomir.wudarczyk@pwr.edu.pl (S.W.); przemyslaw.sperzynski@pwr.edu.pl (P.S.); jacek.balchanowski@pwr.edu.pl (J.B.)

**Keywords:** rehabilitation support, mechatronics, sensors, data acquisition

## Abstract

This article is devoted to the novel method of upper limb rehabilitation support using a dedicated mechatronic system. The mechatronic rehabilitation system’s main advantages are the repeatability of the process and the ability to measure key features and the progress of the therapy. In addition, the assisted therapy standard is the same for each patient. The new method proposed in this article is based on the prediction of the patient’s intentions, understood as the intentions to perform a movement that would be not normally possible due to the patient’s limited motor functions. Determining those intentions is realized based on a comparative analysis of measured kinematic (range of motion, angular velocities, and accelerations) and dynamic parameter values, as well as external loads resulting from the interaction of patients. Appropriate procedures were implemented in the control system, for which verification was conducted via experiments. The aim of the research in the article was to examine whether it is possible to sense the movement intentions of a patient during exercises, using only measured load parameters and kinematic parameters of the movement. In this study, the construction of a mechatronic system prototype equipped with sensory grip to measure the external loads, control algorithms, and the description of experimental studies were presented. The experimental studies of the mechanism were aimed at the verification of the proper operation of the system and were not a clinical trial.

## 1. Introduction

Nowadays, an increasing number and variety of physical activities can be observed in societies around the world. The consequence of this is a significant increase in the number of injuries, which are difficult to avoid even when only taking into consideration normal life and recreational physical activity. Another phenomenon is the ageing of the population, which combined with adverse health-related changes in lifestyle, bring about an increase in the number of strokes, causing, among other issues, upper limb dysfunction. Therefore, the demand for more effective and faster treatment methods has increased.

**Figure 1:**
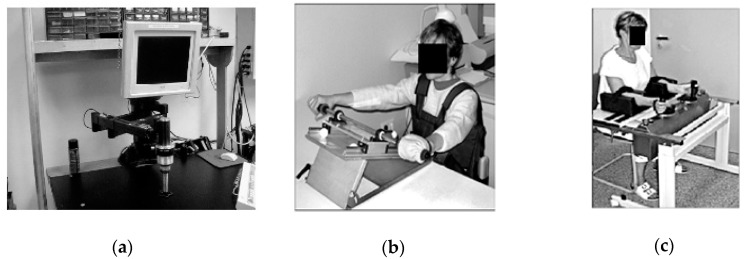
Examples of the first mechatronic rehabilitation supporting devices: (**a**) MIT-MANUS [4], (**b**) Nudelholz [9], (**c**) Bi-Manu-Track [9].

Rehabilitation is one of the most important areas of medicine. It is intended for people who have lost some of their motor functions due to disease or injury or those whose motor functions are limited due to congenital disabilities. Rehabilitation helps to avoid potential complications associated with eventual surgery. The effectiveness of therapy is significantly increased by using dedicated supporting devices, which also contributes to a reduction in treatment time and greatly enhances the final outcome [1,2,3,4,5,6,7,8]. Research concentrating on increasing the effectiveness of upper limb rehabilitation have been carried out in many centers [3], e.g., in Germany in the 1990s [7]. They were based on the pioneering works at Massachusetts Institute of Technology (MIT) (Figure 1a,b) [4,9,10] where, among others, the Bi-Manu-Track system (Figure 1c) was invented and implemented.

All the motion parameters of these systems were chosen individually, and devices recorded the progress of the rehabilitation of the upper limbs. The disadvantage of these solutions was the lack of possibility to achieve a full range of motion of supported movements. Systems called exoskeletons deal with this problem. The axes of rotation of these devices are compatible with the human anatomical axes, and therefore exoskeletons resemble a human limb. Moreover, such a system can be attached to a limb in several places on each limb segment.

**Figure 2:**
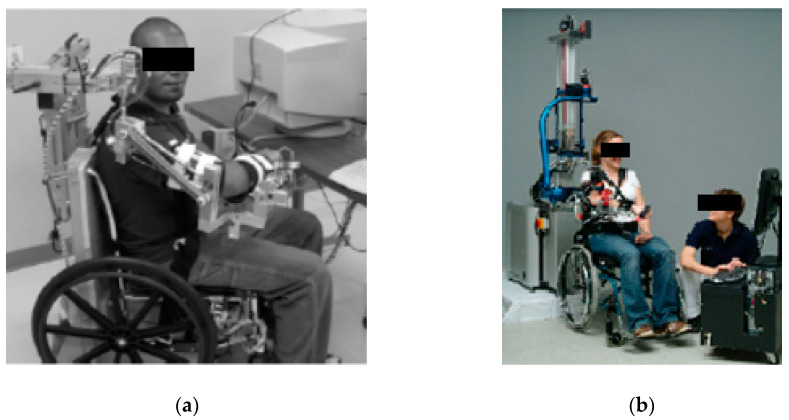
Examples of rehabilitation exoskeletons: (**a**) WREX [11], (**b**) Armin [5].

This results in the better cooperation of the limb with the device. Exoskeleton devices are mainly fixed to a wheelchair (Figure 2a) [11] or the floor (Figure 2b) [1,2,5,12]. The main disadvantage of exoskeleton solutions is the significantly complicated mechanism, which results in their very high production cost. This causes a problem with regards to widespread availability in rehabilitation centers.

Another approach to rehabilitation support is represented by reconfigurable systems, characterized by changeable mobility *W* (in some articles understood as degrees of freedom (DoF) of a kinematic chain). Even with lower mobility (e.g., *W* = 3 [13]), it is possible to perform palmar and dorsal flexion, adduction and abduction of the wrist, and pronation and supination of the forearm. The main advantage of these systems is the ability to fix some kinematic joints so that simple movements can be conducted via a lower number of drives. However, the supporting of complex movements is not possible. Hand movement tracking and supporting systems are also currently being strongly elaborated [12,14,15].

Therapy assisted by the use of rehabilitation devices may eliminate some of the limitations of manual therapy, such as movement repetition quality or progress recording. Placing the patient in a virtual environment allows rehabilitation to be customized to the patient’s individual needs. Many research works confirm the effectiveness of using virtual reality and psychological factors in rehabilitation supporting devices [8].

Rehabilitation is a very long and complex process, and therefore also expensive. It needs engagement and cooperation from both the rehabilitated person and the physiotherapist. What is more, it requires an individual approach to each patient, making the automation of exercises difficult. In all the given examples of devices that support rehabilitation, a subsystem described as mechatronic (technology synergically combining electronics, mechanical engineering and software development) is required [16].

**Figure 3:**
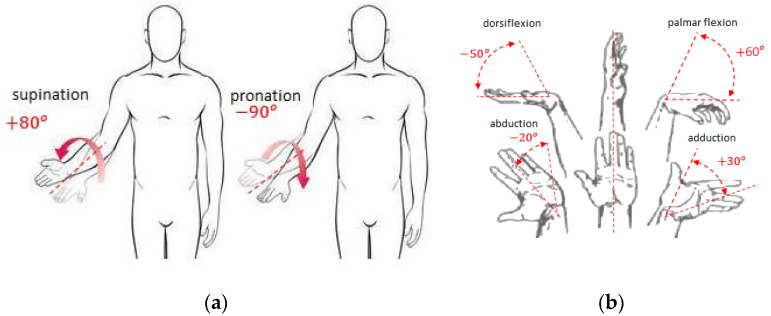
Supported movements of the upper limb with ranges of motion (RoM): (**a**) forearm pronation and supination; (**b**) palmar/dorsiflexion and adduction/abduction of the hand.

In all the above described examples of rehabilitation supporting devices, the treatment movement program was implemented rigidly or manually by the physiotherapist. In the new solution of mechatronic rehabilitation system (MWR) proposed in this work, the patient supervised by the therapist decides on the characteristics of the exercise. The rapid development of sensory techniques, drives, and programming allows extending the functionality of programmed devices that support the rehabilitation process by giving some autonomy to the device. After sensing movement intentions, MWR is to decide whether to support the patient in the movement or hinder the exercise by applying additional load if the patient achieves higher angular velocities of the movement than established by the physiotherapist. The movement intentions are understood as the intention to perform an appropriate supported movement of the upper limb: pronation/supination of forearm; palmar/dorsiflexion; abduction/adduction of the hand (Figure 3). The essence of MWR operation is the ability to measure external loads acting on the device’s grip and at the same time the kinematic parameters of the movement. On this basis, the movement prediction is being made. With properly sensed movement intention, the device will enable proper rehabilitation movements. This is a completely new approach that has the potential for a new quality of treatment.

The main function of the proposed MWR system is to support the rehabilitation process. The paper identifies two cases of using the system in relation to known rehabilitation exercises in order to extend these therapies with measurement and support, and one new method of predicting the patient’s intentions.

The first known exercise is used when the patient’s muscle strength, in states of partial or complete muscle denervation, does not allow for independent movement in a given joint (e.g., in conditions after spinal cord injuries, injuries of the central nervous system, or peripheral nerves—causing paralysis or muscle paresis). In this case, proper passive exercises are essential. These are exercises in which movement is triggered by another person’s appropriate work or a device that moves a part of the patient’s body. Passive exercises are performed in appropriate painless, fully accepted by the patient’s range of motion. The main contraindications for performing passive exercises are:

- acute inflammation of joints and periarticular tissues,
- a lack of full bone union after fracture,
- significantly increased body temperature,
- post-sprain condition,
- the occurrence of pain during exercise.

The second type of proposed supported therapies are active exercises. These refer to the patient’s self-control of muscle tension, which can cause joint movement or conscious muscle tension with the intention to move without performing the exercise (isometric exercises). In this case, the task of the mechanism is to control the movement performed by the patient.

In active exercises, resistance to movement is created by:

- own body weight (active free exercises),
- a therapist who cannot be overcome by the patient (isometric exercises),
- a therapist who hinders the movement of the patient,
- the use of additional weights, rubber bands, expanders (active exercises with resistance),
- specialized mechanisms to support rehabilitation.

As a result of the exercises, the number of shrinking muscle fibers increases, which helps to enhance the efficiency of the work of the muscle pump. An active muscle pump increases the circulation of blood, lymph, and tissue fluids, which improves the nutrition of tissues, and in the case of injury accelerates the healing of damaged tissues. Active exercises involve observing the individual phases of movement: starting position, a movement towards the end position, returning to the starting position. A short break at the end of one repetition of movement improves the blood supply, which in turn supplies nutrients to weakened and damaged tissues, while at the same time accelerating their regeneration. Contraindications to active exercises are:

- the absolute necessity to immobilize parts of the body,
- severe pain sensations,
- acute inflammation of the joints and periarticular tissues,
- when patients are immediately after injuries or operations,
- a significant level of circulatory and respiratory failure,
- when patients are in an unstable or generally severe condition.

The core rehabilitation task is to improve the quality of life of the patient. The greatest negative impact on the quality of life is the inability to perform everyday activities, such as preparing meals, eating, opening the door, and so on. These activities mainly involve three movements: forearm pronation/supination (Figure 3a), and palmar/dorsiflexion and adduction/abduction of the hand (Figure 3b). As a consequence, the mobility W of the MWR that is the subject of this research is equal to three (*W* = 3).

**Figure 4:**
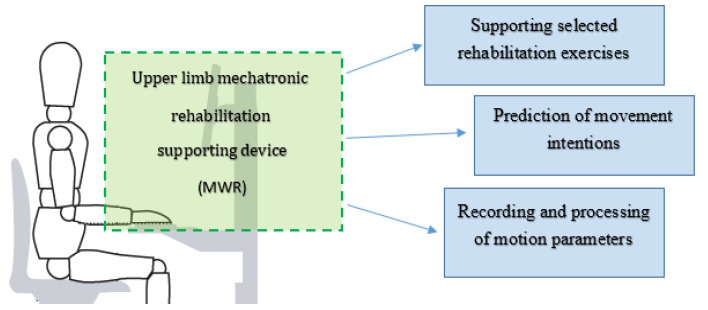
General concept of the novel mechatronic system supporting the rehabilitation of upper limbs.

The general concept of the mechatronic system supporting the rehabilitation of upper limbs (MWR) is presented in Figure 4. The proposed MWR system provides the ability to perform these movements throughout the full range of motion (RoM) for a human limb. In all the examples of devices supporting rehabilitation, a subsystem that can be considered mechatronic is indispensable.

Research on a novel rehabilitation system began in 2014 at the Wroclaw University of Science and Technology [17]. The presented device had a modular structure, and in assumptions it was possible to be used as two separated rehabilitation devices. This conception was developed [18], and a new sensor system tasked with measuring two forces resulting from patient interaction, was added. The mechanical structure of the mechanism was developed using structural and geometrical synthesis methods [19].

The MWR system enables two known rehabilitation exercises to be supported: passive and active. The essential feature in this innovative system is the ability to measure, record and analyze the motion parameters and external loads coming from the patient during exercises. With these features, the device is able to predict the movement intentions of the patient during uncharted exercises, e.g., the movement the patient wants to make at a particular moment.

**Figure 5:**
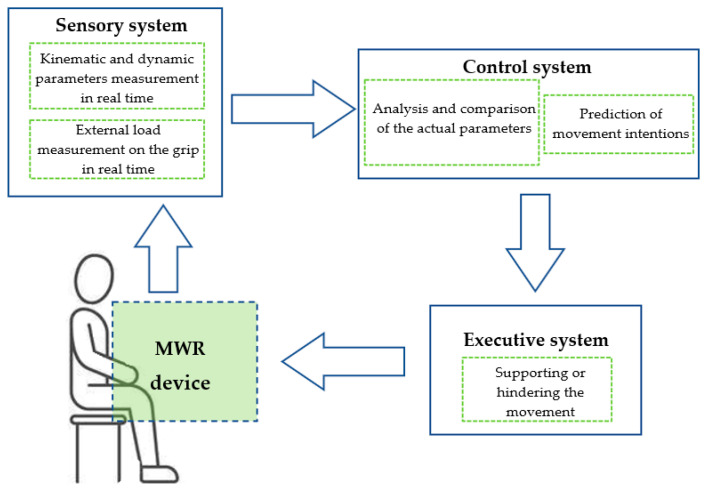
The general concept of predicting movement intentions using the mechatronic rehabilitation system (MWR) mechatronic system.

The prediction program is realized based on a comparative analysis of the measured kinematic and dynamic parameters values, as well as the external loads of the mechanism. Prediction is understood as the calculation of motion parameters based on the external loads on the device’s grip. Then, the control system, knowing the current motion parameters and the patient’s intentions, generates the appropriate control signal to support or hinder the predicted movement (Figure 5). The decision between these two reactions is taken by comparing the actual measured motion parameters and the parameters given by the physiotherapist at the start of the program.

This article presents the basics of the mechanical structure development and operation algorithms of the MWR system with the conducted experimental studies’ results. The aim of the research was the experimental verification of the MWR system operation. Especially, the proper operation of the sensory grip and prediction algorithms.

## 2. Materials and Methods

**Figure 6:**
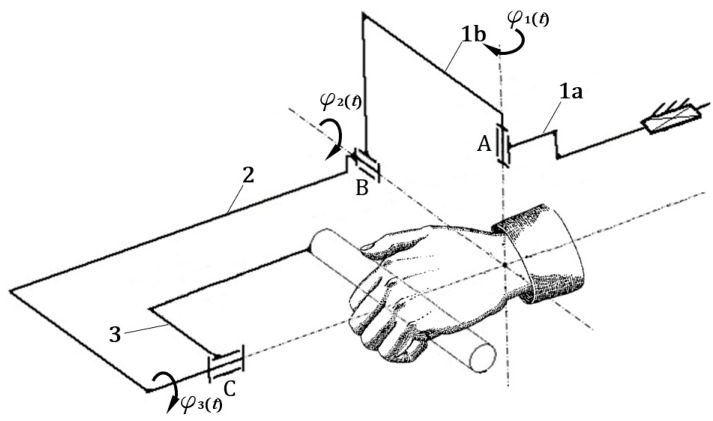
Structure diagram of the second developed mechanism [17].

At first, the authors formed a scheme of the MWR mechanism by type and geometrical synthesis. The system is controlled by three drives $\varphi_{1},$ $\varphi_{2}$, and $\varphi_{3}$, one for each axis. Its main advantage is the ability to support the rehabilitation of the full range of motion of the human upper limb for pronation/supination of the forearm ($\varphi_{3}$), palmar and dorsiflexion of the hand ($\varphi_{2}$), and adduction and abduction of the hand $\left(\varphi_{1}\right)$ (Figure 6).

An indispensable and first step in the complex design process of all mechanisms is the development of their structure. This stage concerns the establishment of an overall solution concept. The task set in this synthesis was to develop a structure, and then a kinematic mechanism scheme, that will support rehabilitation in selected joints of the upper limb. The basic goal of the synthesis was to develop such geometry of the mechanism that would provide the full range of motion in:

- adduction/abduction of the hand: $-20^{{}^{\circ}}<\varphi_{1}\left(t\right)\leq 30^{{}^{\circ}}\left(\Delta \varphi_{1}=50^{{}^{\circ}}\right),$
- palmar/dorsiflexion of the hand: $-50^{{}^{\circ}}<\varphi_{2}\left(t\right)\leq 60^{{}^{\circ}}\left(\Delta \varphi_{2}=110^{{}^{\circ}}\right),$
- $\mathrm{pronation}\;\mathrm{supination}\;\mathrm{of}\;\mathrm{the}\;\mathrm{forearm}:-90^{{}^{\circ}}<\varphi_{3}\left(t\right)\leq 80^{{}^{\circ}}\left(\Delta \varphi_{3}=170^{{}^{\circ}}\right).$

As a result of type and geometrical synthesis, rehabilitation support mechanisms were developed [17], further investigated in the next stage of the work (Figure 6).

**Figure 7:**
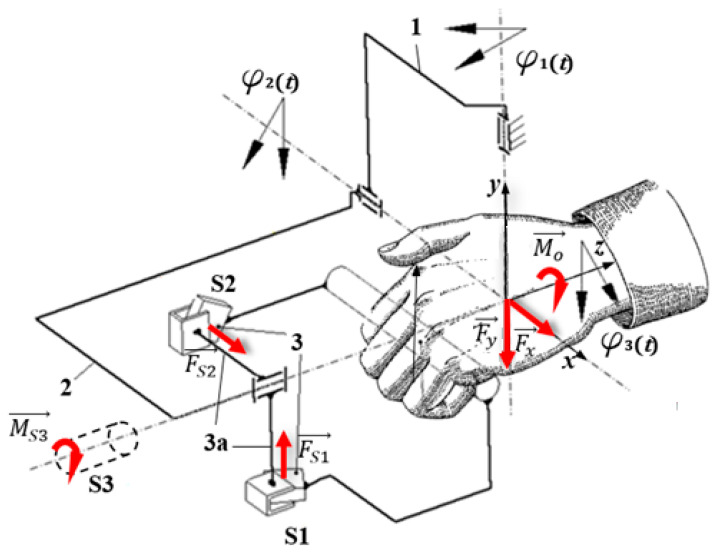
Sensory grip for the MWR mechatronic device—kinematic scheme.

In order to ensure the most accurate measurement of the external loads resulting from a patient’s hand interaction with the MWR device, a new sensor grip (SG) was built. It was designed to measure $F_{x}$ along the grip axis and $F_{y}$ perpendicular load forces, as well as torque $M_{S}$, with which the patient acts on the SG. The special sensors were added do the grip (Figure 7). Force sensor *S*1 and *S*2 form a planar joint between parts 3 and 3a. They are set at right angles to each other. In this situation, during movement in the $\varphi_{2}$ axis, only the *S*1 sensor is loaded (bent), while the *S*2 sensor should barely give any reading. Moreover, only the *S*2 sensor will be loaded during rotation in $\varphi_{1}$. Furthermore, $\varphi_{3}$ torque is measured directly via the *S*3 sensor. With this arrangement of sensors, the external loads $F_{x},F_{y},\;\;M_{o}$, read as $F_{S1},F_{S2}\;\;\mathrm{and}\;M_{S3},$ resulting from the patient’s interaction, can be easily and precisely adjusted to separate supported movements. In this case, reading $F_{S1}$ results from patient interaction during palmar/dorsiflexion movement, $F_{S2}$ during adduction/abduction movement, and $M_{S3}$ results from the forearm’s pronation/supination. As the force sensors *S*1 and *S*2 in the sensory grip SG, two strain gauge beams NA1 were used. The maximum capacity of NA1 beams is within +/− 200 N range with output sensitivity 2.0 mV/V. Moreover, as *S*3 sensor a bidirectional torque NCTE DFM22 series, with nominal torque +/−8.5 Nm was used. NA1 beams were connected to a 4-channel measuring module for load cells ADT4U, while torque sensor was connected directly to the controller.

**Figure 8:**
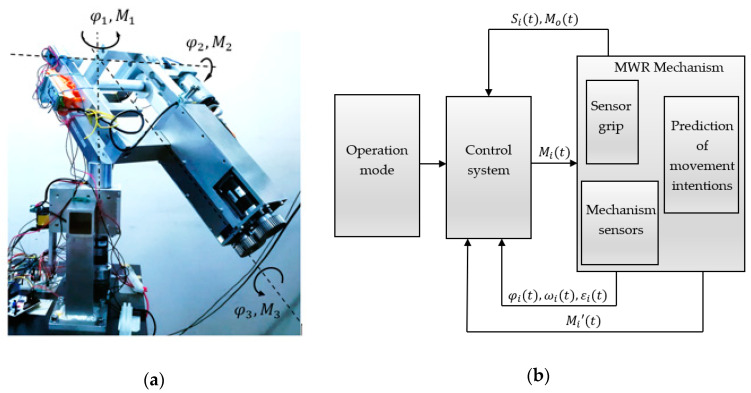
The developed prototype of MWR mechanism: (**a**) view of the MWR prototype; (**b**) control diagram of the MWR mechanism.

The main requirement from the MWR mechanism is to ensure any angular displacement in the range of motion of the selected human joints. The view of the developed prototype is presented in Figure 8a. The scope of this movement is understood as the working space of the device.

**Figure 9:**
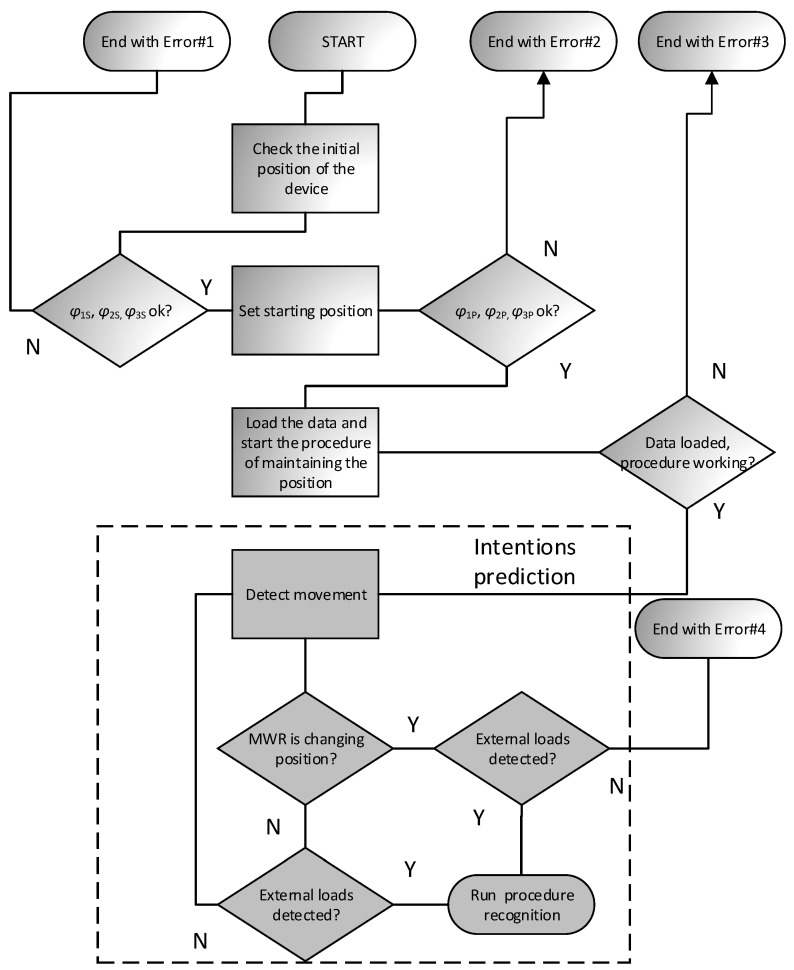
Block diagram of the MWR mechanism operation.

**Figure 10:**
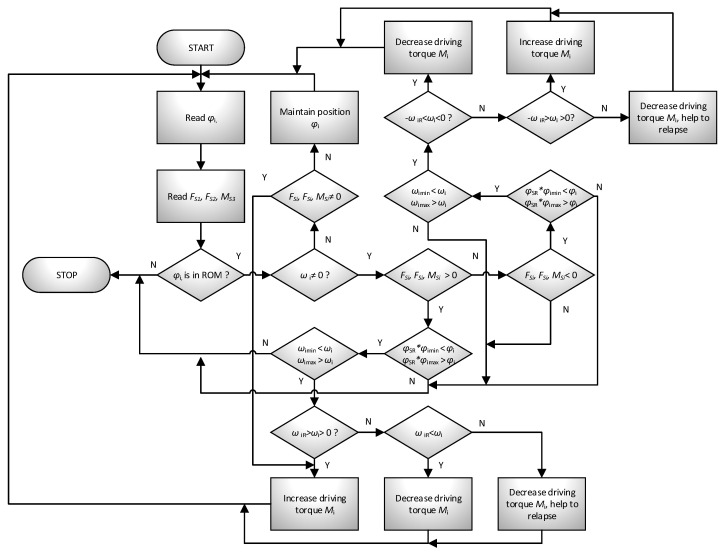
Algorithm of the MWR operation during intentions prediction method.

The system responsible for the operation of the mechanism, and all its decisions and movements, is called the control module. Its task is to excite the movement of active parts by generating appropriate driving moments $M_{1},\;\;M_{2},\;\;M_{3}$ so that it is possible to achieve the set trajectory of the actuator (effector-device grip) with the required accuracy (Figure 8b). The device has a designed response to specific situations according to patients’ intentions. After recognition, the mechanism initiates the appropriate procedure. Five such procedures were implemented in the device: (1) beginning/end of the exercise, (2) maintaining position, (3) supporting/hindering movement, (4) relapse, (5) approaching the range of motion. A general diagram of the control algorithm is shown in Figure 9 and Figure 10.

**Figure 11:**
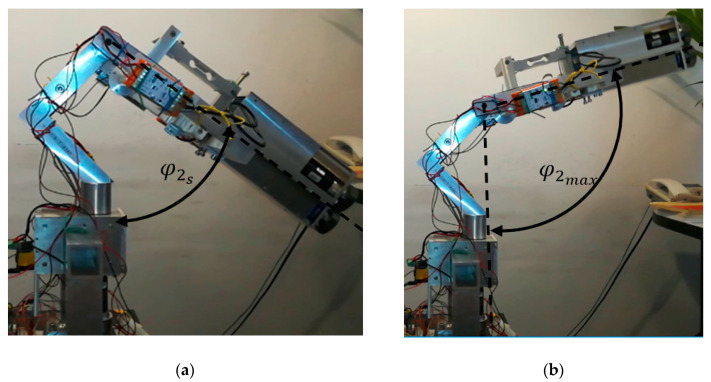
MWR mechanism: (**a**) initial position $\varphi_{2S}$; (**b**) example end position $\varphi_{2max}$.

After starting the system, the position of the mechanism is verified in all axes. The initial position $\varphi_{1S}$ is set at 0°, which corresponds to the anatomical position. The initial position of the third axis $\varphi_{3}$ is set at $\varphi_{3S}=90{}^{\circ}$(maximum supination). The initial position of the second axis $\varphi_{2}$ equals $\varphi_{2S}=13.1{}^{\circ}\;$(Figure 11). Verification is positive if the angular position $\varphi_{1}$, $\varphi_{2}$ and $\varphi_{3}$ does not exceed ±2% of the initial position $\varphi_{1S}$, $\varphi_{2S}$ and $\varphi_{3S}$. Moreover, a physiotherapist must define the following parameters: $\varphi_{imin},\;\varphi_{imax}$—the range of motion for the patient in all i axis, $\omega_{iR}$—the appropriate angular velocity for each axis, $\omega_{imax}$—the maximal angular velocity for each axis. The next step is to set the starting position. It is very important to ensure the safety of the patient during exercise. The main criterion for this phase is setting the starting position $\varphi_{1P}$, $\varphi_{2P}$ and $\varphi_{3P}$, which is either done by the physiotherapist or manually. The condition for a correctly set initial position needs to be within a person’s range of motion (RoM).

If no error occurs, then the position maintenance procedure begins. It involves reading the position of the mechanism $\varphi_{i}$ in real-time and applying the appropriate moment $M_{i}’$ so that the system remains motionless. This allows any (within the range of motion $\varphi_{imin},\;\;\varphi_{imax}$) starting position to be set. The position maintenance procedure will be maintained until the external load is detected on the device handle. If this happens, the system will determine the patient’s movement intentions and then choose to respond to one of three possibilities by implementing the appropriate procedure. The algorithm of predicting patient movement intentions is presented in Figure 10. Generally, it comes down with reading load components $F_{S1},\;\;F_{S2}$ and $M_{S3}$ and refer them to device actual movement, with predefined threshold levels.

The mechanism performs motion support (helps the exercising person to perform the movement) in two situations. In the first case, when the person performing the exercise acts on the handle but does not move the mechanism, $F_{i}\neq 0,\;\omega_{i}=0$. In the second case, when the system is in motion, and the patient wants to relapse. The system helps to overcome the inertia of the parts. The support level is closely related to the movement parameters and the external load reading from the handle (the system compares the read motion parameters $\omega_{i}$ and compares them with the set values $\omega_{iR}$). Additionally, a safety parameter $\varphi_{SR}$, defining the normal working area, was introduced into the system ($0<\varphi_{SR}\leq 1)$. Its modification enables reaching the maximum range of motion to be avoided, e.g., during warm-up. The individual conditions and reactions of the mechanism are presented in Table 1.

Hindering of the movement procedure is realized when the measured motion kinematic and dynamic parameters are greater than the average data $\omega_{iR}$ for the patient. The level of difficulty depends, in the same way as for the assist procedure, on the comparison of motion parameters with the set patterns. The relapse procedure is one of the special procedures. It is activated when the system detects a change in the direction of the force acting on the handle $sgn\left(F_{i}\right)\neq sgn\left(\omega_{i}\right)$.

The procedure of approaching the range of motion belongs to the superior procedures. If the system is brought to a position near the limit of the range of motion, this procedure generates a returning moment in such a way that it prevents the range of motion being exceeded. In extreme cases, it stops the mechanism.

## 3. Results

The experimental research of the MWR device was divided into two parts. The first part consists of sensory grip SG studies, where the assumptions and functionality were examined. The second part consists of experimental research carried out to assess the correct operation of the MWR prototype and to assess the possibility of using the proposed method in the rehabilitation of human hand motor dysfunction.

### 3.1. Experimental Studies of Sensory Grip SG

The first study conducted by the authors concerned the operation of sensory grip SG. Readings from *S*1, *S*2, and *S*3 sensors can be directly assigned to the supported movements of a human upper limb:

- Reading from *S*1 sensor corresponds to the load generated in palmar/dorsiflexion of the hand movement,
- Reading from *S*2 sensor corresponds to the load generated in abduction/adduction of the hand movement,
- Reading from *S*3 sensor corresponds to the load generated in pronation/supination of the forearm movement.

The first verification of the SG assumptions was conducted via simulation studies. A 3D model of the sensory grip was built in MD Adams system. Extensive simulation studies were carried out for various loads and ranges of motion. Examples of simulation research results are presented in this work. Sensors *S*1 and *S*2 were modelled as flexible beam components. The simulation lasting 6 s was divided into 3 parts:

- 0≤t<2s—the grip was loaded only with $F_{y}$ force, simulating dorsiflexion movement,
- 2s≤t<4s—the grip was loaded only with $F_{x}$ force, simulating the abduction movement,
- 4s≤t<6s—the grip was loaded only with $M_{o}$ torque, simulating pronation movement.

**Figure 12:**
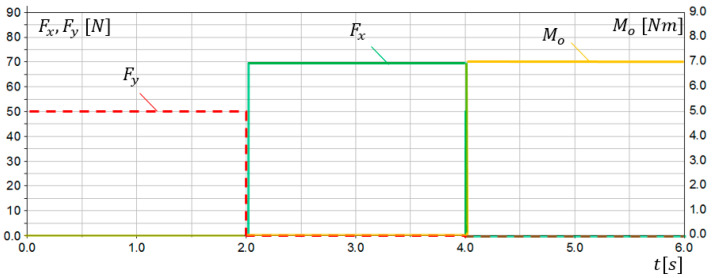
Simulation research results for the sensory grip–characteristics of input loads $F_{y},\;F_{x},\;M_{o}$.

**Figure 13:**
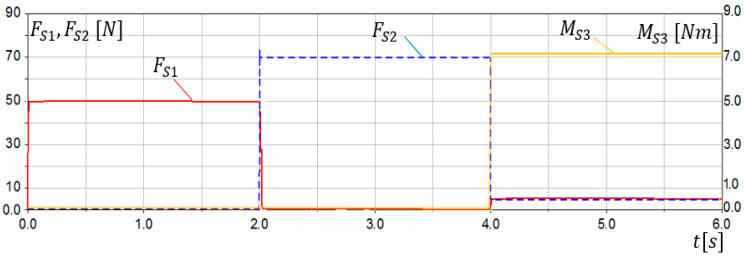
Simulation research results for the grip sensors $F_{S1},\;F_{S2},\;M_{S3}$.

Load characteristics in compliance with Figure 7 are presented in Figure 12. The results of simulation studies $F_{S1}$, $F_{S2}$, $M_{S3}$, as a response on applied loads in *S*1, *S*2, and *S*3 sensors are presented in Figure 13.

The conducted simulation studies confirmed correct operation of the sensory grip. Obtained results indicate that, when SG is loaded with $F_{y}$ force, reading $F_{S1}$ from *S*1 sensor can be observed, while no signal is present at *S*2 and *S*3 sensor. Moreover, when SG is loaded only with $F_{x}$ force, reading $F_{S2}$ from *S*2 sensor can be observed. The torque $M_{o}$ was determined using the sensor *S*3. However non-zero readings $F_{S1}$ and $F_{S2}$ can be observed for 4s≤t<6s, when loads $F_{y}=0$ and $F_{x}=0$. It results from deformation of sensor modelled elements (torsion). Interference with readings $F_{S1}$ and $F_{S2}$ resulting from $M_{o}$ load did not exceed 6% of the $F_{y}$ and $F_{x}$ level. The conducted simulation studies indicated that reading $F_{S1}$ stands for load resulting from palmar or dorsiflexion, while $F_{S2}$ reading can be associated with adduction or abduction movement. On the other hand, $M_{S3}$ readings are related to pronation or supination of the forearm.

**Figure 14:**
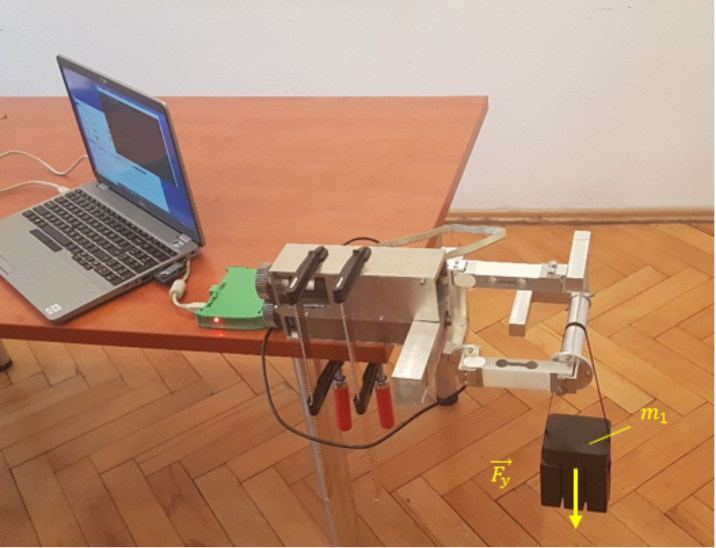
First experimental research of sensor grip (SG): applied $m_{1}=5$ kg load $F_{y}$ in *y* direction.

In the next stage of research, experimental studies of the sensory grip SG were carried out. Three experiments were performed for three load cases:

- the grip was loaded with force $F_{y}=49.05$ N (loaded with $m_{1}$ = 5 kg) (Figure 14),
- the grip was loaded with force $F_{x}=68.67$ N (loaded with $m_{2}$ = 7 kg),
- the grip was loaded with force $M_{o}=7.0$ Nm (the grip was locked in place, load $M_{o}$ was generated by the $M_{3}$ drive).

**Figure 15:**
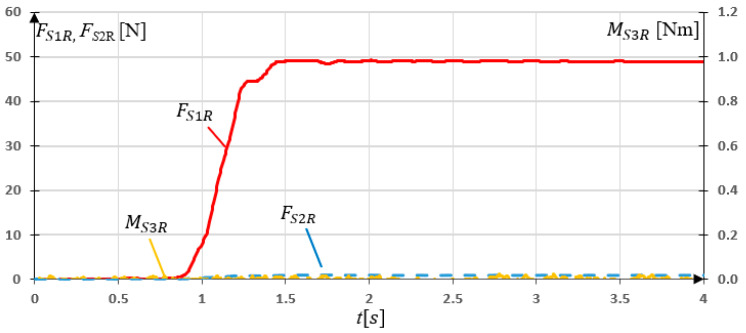
Experimental research results for the grip loaded with 5 kg mass in *y* direction.

The first two experimental research consisted of applying $m_{1}$ or $m_{2}$ mass on the properly oriented sensory grip and measure readings from *S*1, *S*2, and *S*3 sensors. In third research, the grip was locked and $M_{o}$ load was added via $M_{3}$ drive. Measurements were made with a frequency of 50 Hz. Results of experimental research for SG loaded with $F_{y}$ = 49.05 N are presented in Figure 15.

The experimental studies of the sensory grip loaded with $F_{y}$ force showed that almost all the load was read by sensor *S*1. Maximal value of the force $F_{S1R}=49.48$ N. It can be noticed that non-zero values of $F_{S2R}$ readings occurred. The maximal value $F_{S2R}=1.10$ N.

**Figure 16:**
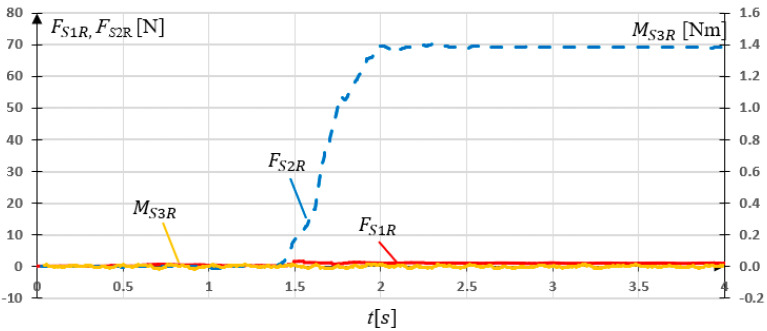
Experimental research results for the grip loaded with 7 kg mass in *x* direction.

In a subsequent study, the grip was rotated $90^{{}^{\circ}}$ and loaded with $m_{2}=7\;\mathrm{kg}$. This corresponds to $F_{x}=68.67$ N force load in *x* direction. The measurement results are shown in Figure 16. The results of the experiment of SG loaded in *x* axis showed the expected data dependencies. *S*2 sensor maximal reading $F_{S2R}=70.25$ N, while non-zero *S*2 reading $F_{S1R}=1.48$ N can be observed.

**Figure 17:**
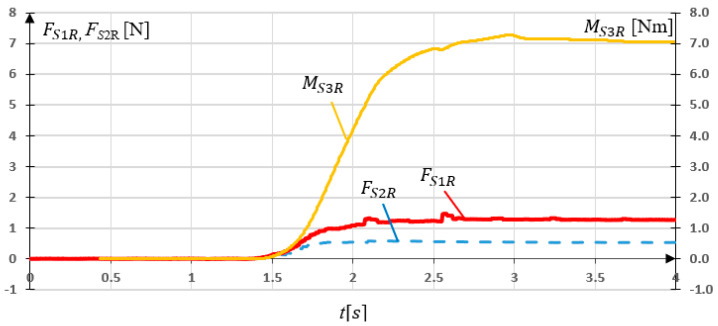
Experimental research results for the grip loaded with 7 Nm torque.

The last research concerning SG consisted on locking the grip in place and generating $M_{3}=7\;\mathrm{Nm}$ torque, which is considered as $M_{o}$ in this research. The experiment consisted in examining the influence of $M_{o}$ on $F_{S1R}$ and $F_{S2R}$ readings. The measurement results are shown in Figure 17.

The $M_{o}$ torque load resulted in $F_{S1R}$ and $F_{S2R}$ readings. The maximal values $F_{S1R}=1.46$ N while $F_{S2R}=0.47$ N occurred. They result from torsion of the sensors (strain gauges) under a given load. The values of these forces are negligible compared to the results of previous research on the SG.

The conducted experimental studies indicated, that reading $F_{S1R}$ stands for load resulting from palmar or dorsiflexion, while $F_{S2R}$ reading can be associated with adduction or abduction movement. On the other hand, $M_{S3R}$ reading is related to pronation or supination of the forearm.

### 3.2. Experimental Research of MWR Mechanism

Experimental studies of the MWR mechanism were carried out in order to assess the correctness of the prototype’s operation and to assess the applicability of the proposed method in the rehabilitation of human hand motor dysfunctions. The study was conducted on a person that does not have any motor dysfunction (male, 30 years old, height 179, mass 83 kg, BMI 25.9). The measurements were carried out with 50 Hz frequency.

**Figure 18:**
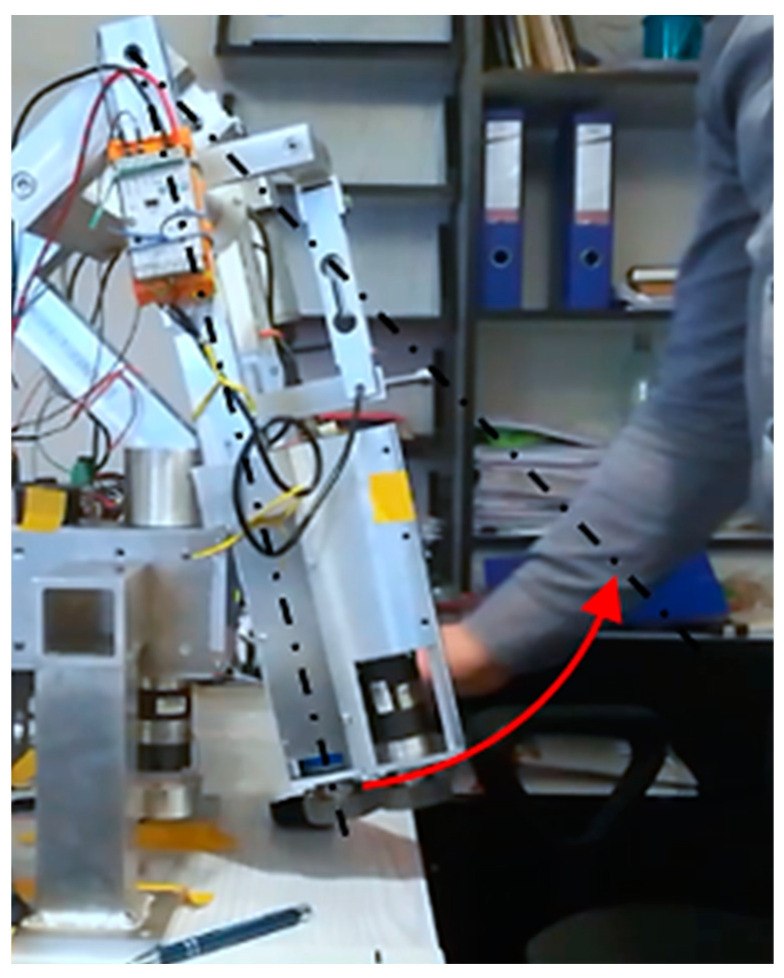
Setting the start position for the $\varphi_{2P}$ axis.

Concerning the device’s mass forces (gravity), the most challenging situation occurs when one on the axis is horizontal. In such a configuration, the greatest influence of mass forces interacts with one driven axis of the device. This is the most extreme load case. For this reason, this article presents experimental studies for the case when the $\varphi_{2}$ axis is horizontal. Research on the MWR mechanism began with manually setting the initial position (Figure 18). The range of motion in this axis is $-55^{{}^{\circ}}\leq \varphi_{2}\leq \;60^{{}^{\circ}}$, where a positive value refers to the hand’s dorsiflexion.

**Figure 19:**
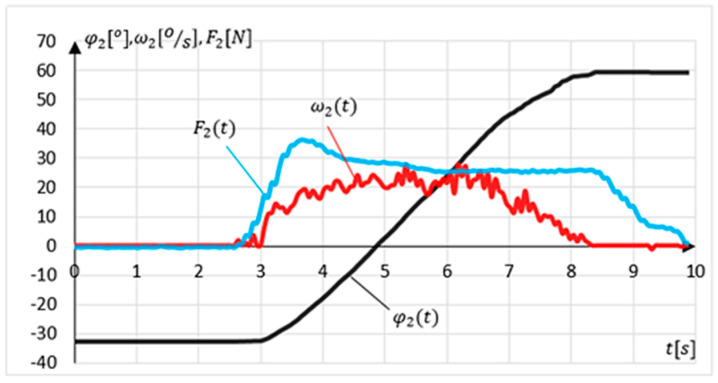
Result of the first experiment-achieving maximum dorsiflexion $\varphi_{2}=\;60^{{}^{\circ}}$.

The first experiment, lasting 10 s, consisted of achieving maximum dorsiflexion $\varphi_{2}=\;60^{{}^{\circ}}$. The mechanism position was set manually, without interaction with the sensory grip, to unknown $\varphi_{2P}$ within RoM. The task of the MWR was to detect the direction of movement, assist the exerciser during acceleration and slow down the device when approaching the RoM. The results of the experiment are shown in Figure 19.

The measured initial position was equal to $\varphi_{2P}=-32.7^{{}^{\circ}}$. In the time interval from 0 s≤t<3 s, the system remained stationary (position retention procedure in operation). At time t=2.7 s, the external load $F_{2}$ appears for the next Δt=0.3 s, and despite the increasing external load value, the mechanism stayed stationary (due to sensitivity level). At that time, the program algorithm detected the dorsiflexion movement’s intention and started assisting, gradually increasing the assisting moment. The maximum external force $F_{2}\;=36.1\;N$ was measured in t=3.6 s. The time error related to of measurement of the force sensor is 0.1 s. The MWR reached its maximal angular position $\varphi_{2}=\;59.6^{{}^{\circ}}$ in t=8.4 s. The maximum end position error $\Delta \varphi_{2}<0.4^{{}^{\circ}}$. Despite the external load in 8.4 s<t≤10 s, the system did not allow the set maximal angular position to be exceeded.

**Figure 20:**
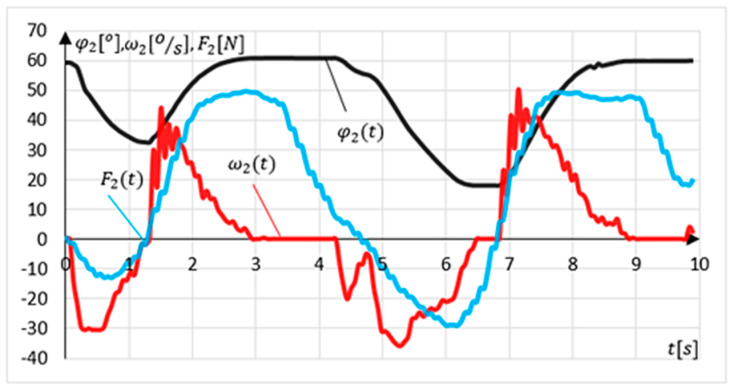
Results of MWR relapse procedure experiments.

Another experiment involved examining the intention to change the movement direction (relapse procedure). The experiment lasted 10 s. The device position was set at maximal dorsiflexion. The exercising person’s task was to make two changes in the direction of movement followed by a return to the starting position. The results of the experiment are shown in Figure 20.

The measured initial position value $\varphi_{2P}=58.7^{{}^{\circ}}$ for this experiment. Relapse is detected if the system is in an angular position outside the values determining the range of motion $\varphi_{2}\leq \;60^{{}^{\circ}}$ and $\varphi_{2}\geq \;-50^{{}^{\circ}}$ in tolerance of ±2%. The relapse procedure is carried out when the system detects a change in the external load direction. In this experiment, there are two such intentions detections at time t=1.26 s and t=6.84 s. The relapse procedure’s side effect is a momentary leap in the angular velocity value $\omega_{2}$ to a maximal value of $\omega_{2}=50.4{}^{\circ}/s$ at time t=6.84 s.

**Figure 21:**
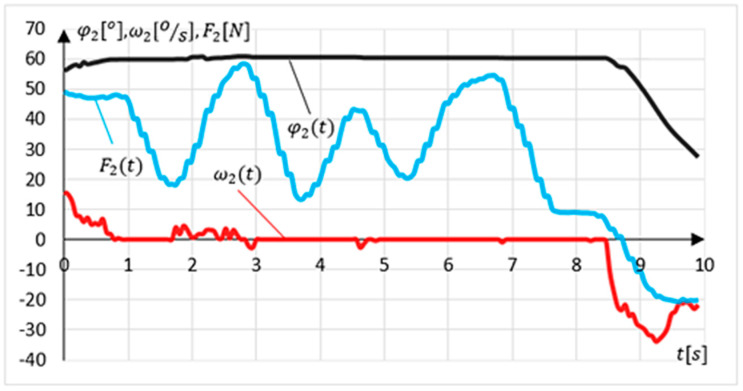
Result of preventing the movement from exceeding the range of motion $\left(\varphi_{2max}=60^{{}^{\circ}}\right)$.

The last experiment was aimed at verifying the procedure of preventing the movement from exceeding the range of motion $\varphi_{2}\leq 60^{{}^{\circ}}$. To that end, the angular position of the device was set near the upper range of motion. The task of the exercising person was to try to exceed the ROM three times. To make this experiment possible, the hand during the exercises was free. The results of the experiment are shown in Figure 21.

During this experiment, the measured value $\varphi_{2P}=56.9^{{}^{\circ}}$. Three pulsed attempts to exceed the range of motion were detected at $t_{1}=2.88\;s,\;t_{2}=4.62\;s,\;\mathrm{and}\;t_{3}=6.66\;s$. The greatest force $F_{2}=58.0$ N was measured during the first attempt. During this test, a slight deviation of the position ($\Delta \varphi_{2}=0.87^{{}^{\circ}}$) was observed. During the remaining attempts, no change in the angular position of the mechanism was observed.

## 4. Discussion

The authors developed a sensor system and software in such a way that the maximum response time of the mechanism was 0.12 s. This response time did not disturb the patient during exercises. This time is related to the averaging of the force sensors readings measured in the grip. It is a compromise between maintaining the appropriate measurement accuracy and mechanism response time. It is planned to test a system and a higher frequency of measurement, which will allow to reduce the response time of the system to 0.05 s.

The disturbances $F_{S2R}$ at Figure 15 and $F_{S1R}$ at Figure 16 are caused by the friction force in the planar joint mounting of *S*1 and *S*2 sensor. However, the values of these forces are negligible.

When a patient suffers some motor dysfunctions, the organism tries to compensate it using other motor units. Therefore, impacts and sudden pulls with the use of body weight may appear. For this reason, the sensor system must be protected. The MWR structure may seem oversized, however, it is designed to protect both the patient and sensors. The authors are currently working on optimization of the MWR mass and structure. The authors estimate that it is possible to reduce inertia impact of the device for about 30%.

Supporting the rehabilitation process can significantly increase the results of therapy and patient comfort. Any dysfunction of the upper limb can lead to a significant deterioration in a person’s quality of life. In the course of experimental research, compliance with the assumptions and correctness of system operation was demonstrated.

Many studies regarding joint loads in human hand were carried out [20] especially in sports like golf [21] or volleyball [22], upper limb pathologies research works [2], or kinematic parameters measurement systems [14]. Still, there is a lack of information on the healthy person performing reaching RoM tasks, and forces during normal functionality movements. Robotics assisted loads can be found in other sources. Average joint torques in the elbow and wrist are approximately one-tenth and one-hundredth, respectively, of those experienced at the shoulder, with median torques at the shoulder ranging from 0.4 to 4 Nm [23]. Reach to grasp movements are also measure in literature, however using EMG method, at peak reaching about 16 N [24]. Considering the order of magnitude, the achieved in this article values are correct. The provided results of human joint loads might be valuable for psychotherapists.

The research results presented in the work prove the correct functioning of the MWR system and sensing movement intentions method. Moreover, they prove the safety of the exercises themselves. The presented prototype does not allow to exceed the RoM, which was proved in the third experiment.

The proposed method can help patients without severe paresis. The authors expect that the possibility of “independent” decisions about the movement, with the supervision of a physiotherapist, may positively impact the rehabilitation process. When designing the device, the authors also took into account the economic aspects, having in mind that the device should be accessible to a wide group of patients.

## 5. Conclusions

The prediction of movement intention is a new method of rehabilitation support which holds great potential. Not only does it allow for customization of the load to the individual patient needs, but it also does not need to know the specific exercise. The patient can decide what action he wants to perform by himself. The device provides comfort, safety and recorded information for the physiotherapist to analyze. In addition, it is possible to assess the effectiveness of specific therapies due to the possibility of comparing numerical results.

In further work, the authors plan to examine the developed method of predicting patient intentions on a specific group of patients in order to assess its effectiveness in relation to specific dysfunctions or diseases.

## Figures and Tables

**Table 1:** Simplified principle of the prediction mode.

Control Signal	Status of the Device		Procedure
$F_{i}=0$	$\omega_{i}=0$	$\varphi_{SR}*\varphi_{imin}<\varphi_{i}$ $\varphi_{SR}*\varphi_{imax}>\varphi_{i}$ $\omega_{imax}>\omega_{i}$	Maintain position
$F_{i}>0$	$\omega_{iR}>\omega_{i}>0$		Assist movement proportionally
	$\omega_{i}<0$		Relapse procedure
	$\omega_{iR}<\omega_{i}$		Hinder movement proportionally
	$\omega_{iR}>\omega_{i}>0$	$\varphi_{i}\geq \varphi_{SR}*\varphi_{imax}$	Approaching the range of motion
$F_{i}<0$	$-\omega_{iR}<\omega_{i}<0$	$\varphi_{SR}*\varphi_{imin}<\varphi_{i}$ $\varphi_{SR}*\varphi_{imax}>\varphi_{i}$ $\omega_{imin}>\omega_{i}$	Assist movement proportionally
	$\omega_{i}>0$		Relapse procedure
	$-\omega_{iR}>\omega_{i}$		Hinder movement proportionally
	$-\omega_{iR}<\omega_{i}<0$	$\varphi_{i}\leq \varphi_{SR}*\varphi_{imin}$	Approaching the range of motion

## Data Availability

Data is contained within this article.
